# Diagnostic Usefulness of IFN-Gamma Releasing Assays Compared With Conventional Tests in Patients With Disseminated Tuberculosis

**DOI:** 10.1097/MD.0000000000001094

**Published:** 2015-07-17

**Authors:** Shi Nae Yu, Jiwon Jung, Yong-Kyun Kim, Ju Young Lee, Sun-Mi Kim, Su Jin Park, Sang-Oh Lee, Sang-Ho Choi, Yang Soo Kim, Jun Hee Woo, Sung-Han Kim

**Affiliations:** From the Department of Infectious Diseases, Asan Medical Center, University of Ulsan College of Medicine, Seoul (SNY, JJ, Y-KK, JYL, S-MK, SJP, S-OL, S-HC, YSK, JHW, S-HK); and Department of Infectious Diseases, Soonchunhyang University Cheonan Hospital, Cheonan, Republic of Korea (SNY).

## Abstract

IFN-gamma releasing assays (IGRAs) such as T-SPOT.*TB* assay and QuantiFERON-TB In-Tube (QFT-GIT) have yielded promising results for the diagnosis of tuberculosis (TB). However, little is known about the usefulness of these assays for diagnosing disseminated TB. We therefore compared their usefulness with traditional tests in patients with disseminated TB. All adult patients with suspected disseminated TB were prospectively enrolled at a tertiary hospital in an intermediate TB-burden country during a 6-year period. Disseminated TB was defined as involvement of the bone marrow or ≥2 noncontiguous organs, or presence of miliary lung lesions. A total of 101 patients with confirmed and probable disseminated TB were finally analyzed. Of these 101 patients, 52 (52%) had miliary TB and the remaining 49 (48%) had nonmiliary disseminated TB. In addition, 63 (62%) had no underlying disease. Chronic granuloma with/without necrosis, acid-fast bacillus staining, *Mycobacterium tuberculosis* PCR, and culture for *M tuberculosis* were positive in 77% (41/53), 43% (43/101), 70% (67/96), and 72% (73/101), of the patients, respectively. The T-SPOT.*TB* assay was positive in 90% (91/101) of them. The sensitivity of the T-SPOT.*TB* assay in patients with miliary TB (90%) was similar to that in patients with nonmiliary TB (90%) (*P* > 0.99). In a subgroup analysis of the 58 patients in whom both QFT-GIT and the T-SPOT.*TB* results were available, the sensitivity of QFT-GIT (67%) was lower than that of T-SPOT.*TB* (95%) (*P* < 0.001).

In conclusion, T-SPOT.TB assay may be a helpful adjunct test for disseminated TB.

## INTRODUCTION

Disseminated tuberculosis (TB) that involves 2 or more noncontiguous sites is a life-threatening form of TB^1–3^ and accounts for about 5% of all cases.^3^ It mimics a variety of diseases, and delayed diagnosis and initiation of therapy are associated with high mortality.^3^ Hence, a high index of suspicion for disseminated TB is needed. While invasive diagnostic procedures involving various sites such as liver and bone marrow have relatively high sensitivities for diagnosis of disseminated TB,^2,3^ these invasive procedures are often excluded by the critical condition of the patients. The sensitivities of mycobacterial cultures from various suspicious sites are reported to be 20–100%,^3^ but culture can take 2 to 6 weeks and often delays diagnosis and the initiation of therapy. Among noninvasive rapid diagnostic tests whose results are available within 3–5 days, the sputum acid-fast bacillus (AFB) smear, the *Mycobacterium tuberculosis* polymerase chain reaction (PCR), and the tuberculin skin test (TST) have reported sensitivities of 61%, 79%, and 61%, respectively,^4,5^ which are unacceptably low in these critically ill patients. Therefore, a more sensitive noninvasive diagnostic test is urgently needed to guide the immediate initiation of antituberculous treatment.

Recently, IFN-gamma releasing assays (IGRAs) such as the T-SPOT.*TB* assay and QuantiFERON-TB in-tube (QFT-GIT) have given promising results for diagnosis of latent TB infection^6^ and active TB.^7–9^ Our group has also demonstrated that the T-SPOT.*TB* assay may be a useful adjunctive test for various types of extrapulmonary TB.^10–13^ However, little is known about the usefulness of these assays for diagnosing disseminated TB. We therefore compared their usefulness with traditional tests in patients with disseminated TB.

## METHODS

### Study Population

All adult patients with suspected disseminated TB were prospectively enrolled at the Asan Medical Center, a 2700-bed tertiary hospital in Seoul, South Korea, between March 2008 and December 2013. Patients were included if they had any clinical symptoms, signs, or radiographic evidence of suspected disseminated TB; there were no exclusion criteria. Microbiological and pathological specimens for diagnosis of disseminated TB were processed by standard techniques and procedures, as described previously.^10–13^ The study protocol was approved by the Institutional Review Board of our hospital.

### Definitions

Disseminated TB was defined as isolation of *M tuberculosis*, positive PCR, or histologic demonstration of caseating granulomatous inflammation from blood, bone marrow, liver biopsy, or at least 2 noncontiguous organs, with/without miliary lung lesions.^2,3^ The clinical categories of patients with disseminated TB have been described previously.^10–12^ Briefly, patients classified as having confirmed TB were those with clinical specimens positive for *M tuberculosis* by culture or PCR assay. Patients were classified as having probable TB if histopathologic examination of biopsy samples showed caseating granuloma and there was a good response to antituberculous therapy. Immunocompromised patients were defined as those with underlying diseases such as HIV infection, malignancy, liver cirrhosis, and chronic renal failure, or those receiving immune suppressive treatment.^13^

### IGRAs

The T-SPOT.*TB* test (Oxford Immunotec, Abingdon, UK) was performed in the research laboratory of our department as described previously.^10–13^ Briefly, a peripheral venous blood sample was collected from each patient for the ELISPOT assay testing for T-cell responses leading to interferon-γ production. Peripheral blood mononuclear cells (PBMCs) were isolated, and 2.5 × 10^5^ PBMCs were plated per well in wells precoated with anti-human interferon-γ antibody. The PBMC were cultured in the well at 37 °C for 18 hours, the assay was performed and spots were counted with an automated microscope (ELiSpot 04 HR; Autoimmune Diagnostika GmbH, Strassberg, Germany). The criteria for positive, negative, and indeterminate outcomes were those recommended by the manufacturer. The outcome was considered indeterminate if the number of spots in the positive control well was < 20 (low mitogen response) or the number of spots in the negative control well was >10 (high nil response).

QFT-GIT (Cellestis, Carnegie, Victoria, Australia) has been performed in the routine clinical laboratory of our hospital since 2010.^14,15^ Consequently, QFT-GIT was recommended for patients with suspected disseminated TB in our routine clinical practice. The detailed procedure was as follows. A peripheral venous blood sample was placed directly into three 1 mL tubes containing, respectively, first, mycobacterium tuberculosis early secreted antigenic target of 6 kDa (ESAT-6), culture filtrate protein 10 (CFP-10) and TB 7.7, second, phyto-hemagglutinin (a mitogen used as a positive control), and third, saline (nil used as a negative control). The samples were incubated at 37°C for 16–18 h, then processed and tested for quantitative interferon-γ levels (IU/mL). The assay was interpreted according to the manufacturer's instructions.

### Statistical Analyses

Statistical Analyses were performed with SPSS for Windows (Version 18.0K; SPSS Inc, Chicago, IL). Categorical variables were compared using Pearson *χ*^2^ test or Fisher exact test, as appropriate. Continuous variables were compared with Student *t* test or the Mann–Whitney *U* test, as appropriate. All tests of significance were two-tailed, and *P* ≤ 0.05 was considered statistically significant. Diagnostic performance was expressed as sensitivity, specificity, positive predictive value, and negative predictive value.

## RESULTS

### Patient Characteristics

A total of 118 patients with suspected disseminated TB were enrolled during the study period. Of these, 101 (86%) patients were classified as disseminated TB comprising 87 (86%) confirmed and 15 (14%) probable cases of disseminated TB, while the remaining 17 (14%) were classified as not-TB comprising 4 malignant tumors, 4 metastatic bacterial infections, 4 connective tissue diseases, 1 cryptococcosis, and 4 other diseases. Of the 101 patients with disseminated TB, 52 (52%) had disseminated TB with miliary lung lesions (miliary TB) and the remaining 49 (48%) had disseminated TB without miliary lung lesions (non-miliary disseminated TB). Baseline clinical characteristics of these 2 groups are shown in Table 1. The most common comorbid condition was diabetes mellitus (10%). Twenty-nine (29%) patients including 6 HIV-infected patients had immunosuppressive conditions, and 63 (62%) had no underlying disease. The most frequently involved organs other than the lung were lymph nodes (36%), followed by the central nervous system (31%). Of the total of 69 cases in which drug susceptibility tests were performed, resistance to at least 1 anti-TB drug was found in 6 (9%) cases, and multidrug resistance, in which TB was resistant to at least isoniazid and rifampin, was found in 2 others (3%). All of the 101 patients were given the T-SPOT.*TB* assay, and 58 the QFT-GIT assay.

**TABLE 1:**
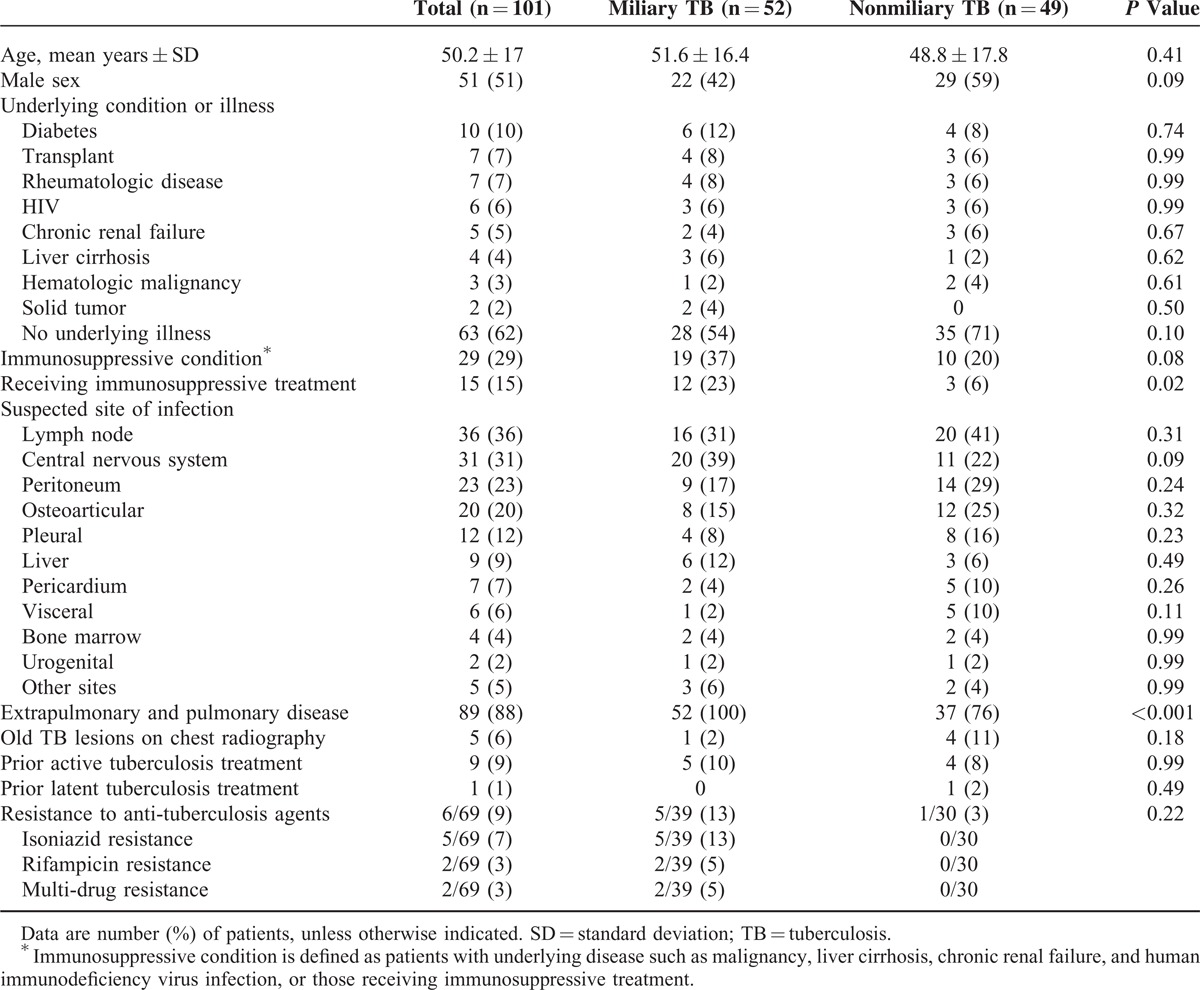
Baseline Characteristics of 101 Patients With Disseminated Tuberculosis

### Results of the Diagnostic Tests

The results of mycobacterial culture of the various specimens are shown in Table 2. The most frequent specimen tested was sputum, followed by cerebrospinal fluid. The overall sensitivity of mycobacterial culture from the various specimens was 53%. The histopathological findings for the 58 patients whose biopsy specimens were available are shown in Table 3. The overall sensitivity of the presence of granuloma with or without necrosis was 81%. The diagnostic yields of conventional diagnostic tests and IGRAs are summarized in Table 4. Chronic granuloma with/without necrosis, the acid-fast bacillus stain, *M tuberculosis* PCR, and culture for *M tuberculosis* were positive in 77% (41/53), 43% (43/101), 70% (67/96), and 72% (73/101) of the patients, respectively. The T-SPOT.*TB* assay was positive in 90% (91/101). QFT-GIT was given to 58 patients, and 39 samples were positive (67%). The tuberculin skin test had lower sensitivity in patients with miliary TB (25%) than in those with non-miliary TB (63%) (*P *= 0.002), while the sensitivity of the T-SPOT.*TB* assay in patients with miliary TB (90%) was similar to that in those with non-miliary TB (90%) (*P* > 0.99). In a subgroup analysis of the 58 patients in whom both QFT-GIT and the T-SPOT.*TB* were available, the sensitivity of QFT-GIT (67% [39/58]) was lower than that of the T-SPOT.*TB* (95% [55/58]) (*P < *0.001) (Table 4). When we analyzed all patients with suspected disseminated TB to calculate the diagnostic performance of T-SPOT.*TB* assay, sensitivity, specificity, positive predictive value, negative predictive value were 90% ([91/101] 95% CI 83–95%), 53% ([9/17] 95% CI 28–77%), 92% (95% CI 85–96%), and 47% (95% CI 24–71%), respectively.

**TABLE 2:**
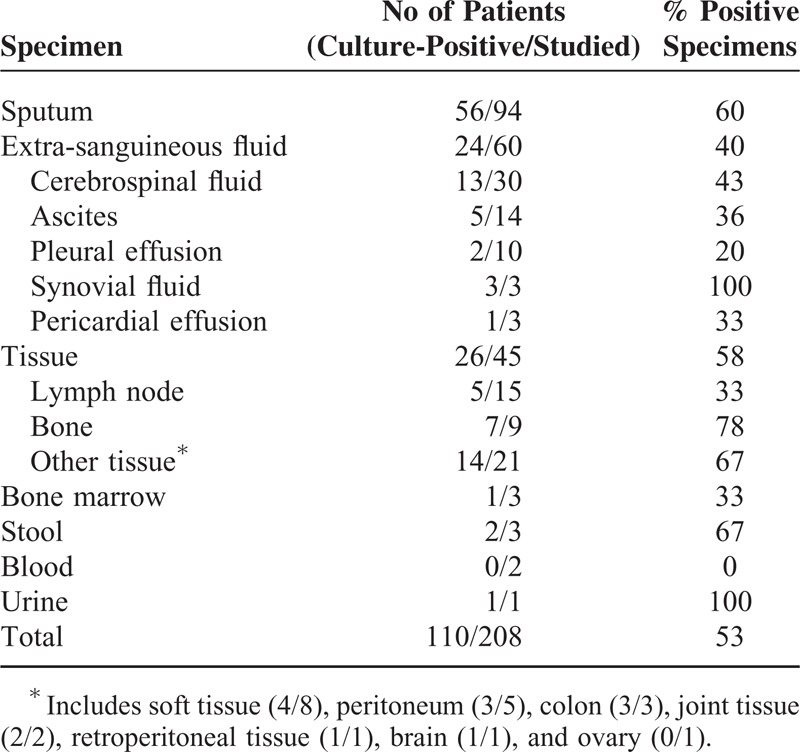
Results of Mycobacterial Culture for 101 Patients with Disseminated Tuberculosis

**TABLE 3:**
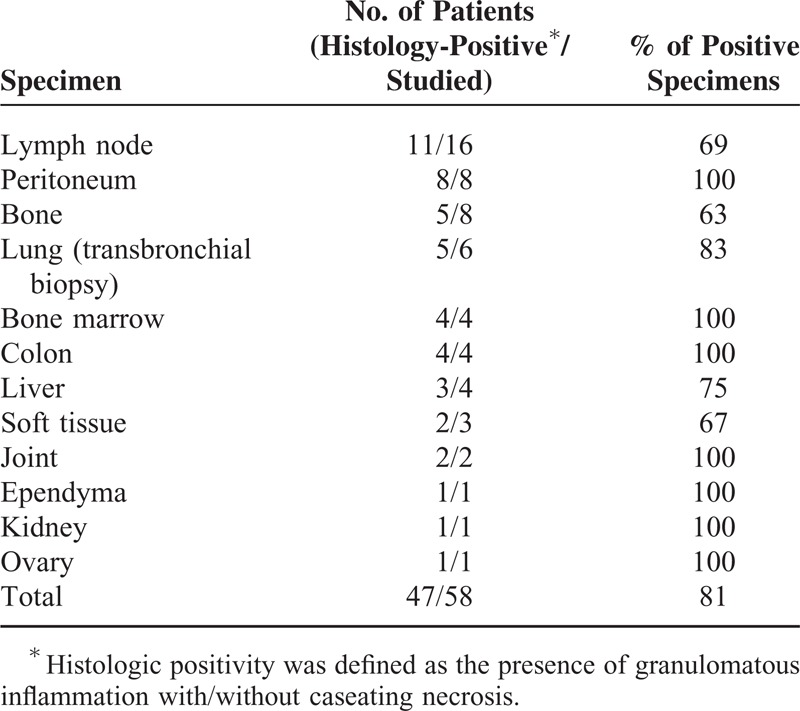
Histolopathologic Findings in 101 Patients With Disseminated Tuberculosis

**TABLE 4:**
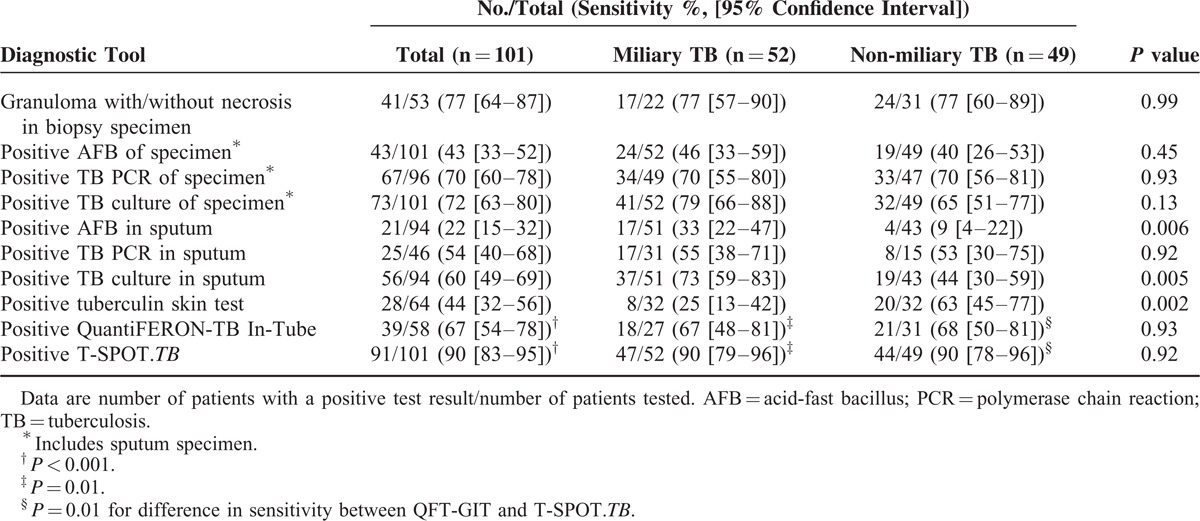
Results of Diagnostic Tests for Disseminated Tuberculosis

## DISCUSSION

Rapid diagnosis and treatment are important prognostic factors for patients with disseminated TB.^1–3^ However, the major problem associated with this form of TB is the difficulty in diagnosing infection early enough to be of value in patient management. A definitive diagnosis of disseminated TB can be made by isolating and identifying *M tuberculosis* in clinical specimens such as secretions or biopsy tissues. A previous study found that the sensitivity of *M tuberculosis* sputum culture was 97%, which appeared to be high enough to rule out the diagnosis.^3^ However, in the present study the sensitivity of *M tuberculosis* sputum culture was as low as 60%. The reason for this discrepancy is not clear because similar proportions of miliary TB were included in the 2 studies (47% in the previous study and 52% in the present one). However, since a previous study of miliary TB^4^ reported that the sensitivity of *M tuberculosis* sputum culture was 77%, which is similar to our figure (73% in miliary TB), it is sensible at the present time to assume that a negative result in the *M tuberculosis* sputum culture does not rule out disseminated TB. In addition, it usually takes at least 2–4 weeks to obtain the results of *M tuberculosis* culture, and this often delays diagnosis and the initiation of therapy.

Histopathological examination of biopsy specimens obtained by invasive procedures can provide rapid and sensitive results. However, obtaining a tissue biopsy sample is not without risk in a patient with suspected disseminated TB. Furthermore, because of the lack of rapid, sensitive, noninvasive tests, it is often unclear whether to perform an invasive test or wait for the response to anti-tuberculous treatment, particularly in countries with a high TB burden. The conventional rapid, noninvasive diagnostic tests have low sensitivity in the case of disseminated TB. The sensitivities of the sputum AFB stain and sputum *M tuberculosis* PCR in patients with disseminated TB were less than 60–80%. In this problematic clinical context, our study demonstrates that, of the noninvasive rapid diagnostic tests whose results are available within 3–5 days, IGRAs, especially the T-SPOT.*TB,* have the highest sensitivities (90%). Therefore, the T-SPOT.*TB* assay may be a useful adjunctive diagnostic tool for disseminated TB.

In a previous study, we found that the sensitivity of the T-SPOT.*TB* in 43 patients with miliary TB was as high as 93%.^16^ On the other hand, Kim et al reported the sensitivity of QFT-GIT in patients with miliary tuberculosis was to be 68% (95% CI 46–97), which is suboptimal for this critically ill patient group.^17^ Of the 101 patients in our cohort, 58 who were enrolled since 2010 underwent QFT-GIT in a clinical laboratory as well as the T-SPOT.*TB* in our research laboratory. Our analysis revealed that the sensitivity of QFT-GIT (67%) was lower than that of T-SPOT.*TB* (95%) (*P* value < 0.001). We suppose that the net responsiveness to IGRAs is determined by antigenic load and host immune response. Antigenic load may be higher in disseminated TB and mycobacterial culture-positive TB than in nondisseminated TB and mycobacterial culture-negative TB. The host immune response may depend on how long mycobacterial antigens have stimulated the host immune system, and how much the host immune system is suppressed by some drug or by the TB itself. Thus, responses to IGRAs are the net outcomes of a complex and continuous interplay of host immune responses to TB antigens. In this regard, we assume that the difference in methodology between ELISPOT-based assays (ie, the T-SPOT.*TB*) and ELISA-based assays (ie, QFT-GIT) may be responsible for the different thresholds of these tests. Thus, the sensitivity to the T-SPOT.*TB* is strongly affected by the antigenic load with only a slight effect of immunosuppressed status, as shown in our previous work^18,19^ where we failed to identify either immunosuppression or lymphopenia as risk factors for false-negative T-SPOT.*TB* tests. In contrast, the sensitivity to QFT-GIT is strongly affected by immunosuppressed status with only a slight effect of antigenic load, as supported by the finding^20^ that both immunosuppression and lymphopenia are independent risk factors for QFT-GIT. Until a large prospective study comparing these 2 commercially available assays in patients with disseminated tuberculosis is available, it will be prudent to use the T-SPOT.*TB* assay as an adjunct for diagnosing this form of TB.

Our study has a few potential limitations. First, it was conducted in a single center on a small number of patients with disseminated TB, although it is the largest study so far to systematically evaluate the diagnostic performance of IGRAs in such patients. Second, it included only a small number of HIV-infected patients (6%), so it cannot be extrapolated to HIV-infected patients. It will be important to establish whether IGRAs retain their high sensitivity in those patients. Third, in South Korea where is intermediate prevalence of tuberculosis, there have been no indigenous cases of endemic fungi such as coccidiosis and histoplasmosis which may produce similar clinical and histopathologic features. Another consideration is that the specificity of the IGRA for active tuberculosis depends on the prevalence of latent TB infection in the population studies because the immunodiagnosis of TB detects active TB and latent TB infection.^10^ In this context, the diagnostic performance in this study should be cautiously interpreted because the prevalence of disseminated TB-mimicking diseases and latent TB infection is various between regions. Additional studies including such controls are needed to define the diagnostic flow of such patients with suspicious disseminated TB with or without miliary lung nodules.

In conclusion, the ELISPOT assay may be a helpful adjunct test for disseminated TB. Further work is needed to investigate the diagnostic performance of the IGRAs in these patients.
